# Tribocorrosion Failure Mechanism of TiN/SiO*_x_* Duplex Coating Deposited on AISI304 Stainless Steel

**DOI:** 10.3390/ma9120963

**Published:** 2016-11-26

**Authors:** Qiang Chen, Zhiwen Xie, Tian Chen, Feng Gong

**Affiliations:** 1 Southwest Technology and Engineering Research Institute, Chongqing 400039, China; 2009chenqiang@163.com; 2 School of Mechanical Engineering and Automation, University of Science and Technology Liaoning, Anshan 114051, China; 3 Chongqing Institute of Green and Intelligent Technology, Chinese Academy of Sciences, Chongqing 400714, China; tianchen@cigit.ac.cn; 4 Guangdong Provincial Key Laboratory of Micro/Nano Optomechatronics Engineering, Shenzhen University, Shenzhen 518060, China

**Keywords:** TiN/SiO*_x_* duplex coating, corrosion, tribocorrosion, force-corrosion synergistic effect, multi-degradation failure

## Abstract

TiN/SiO*_x_* duplex coatings were synthesized on AISI304 stainless steel by plasma immersion ion implantation and deposition (PIIID) followed by radio frequency magnetron sputtering (RFMS). The microstructure and tribocorrosion failure behaviors of the duplex coatings were investigated by X-ray diffraction, X-ray photoelectron spectroscopy, scanning electron microscopy, atomic force microscopy, reciprocating-sliding tribometer, and electrochemical tests. The as-deposited duplex coating had a two-layered columnar growth structure consisting of face-centered cubic TiN and amorphous SiO*_x_*. Sliding tests showed that the TiN interlayer had good adhesion with the substrate, but the SiO*_x_* layer suffered from severe delamination failure. Friction force induced a number of micro-cracks in the coating, which provided channels for the diffusion of NaCl solution. The tribocorrosion test showed that the duplex coating exhibited a lower wear-performance in NaCl solution than in ambient atmosphere. Multi-scale chloride ion corrosion occurred simultaneously and substantially degraded the bonding strength of the columnar crystals or neighboring layers. Force-corrosion synergy damage eventually led to multi-degradation failure of the duplex coating. The presented results provide a comprehensive understanding of the tribocorrosion failure mechanism in coatings with duplex architecture.

## 1. Introduction

In view of its low cost, ease of manufacturing, excellent mechanical properties, and corrosion resistance, stainless steel is the most popularly used material in artificial implants [1,2,3]. However, the synergistic effect of severe chloride ion corrosion and wear-corrosion greatly affects the reliability and service life of stainless steel in chloride ion-rich corrosive media [4,5,6,7]. Therefore, it is necessary to develop protective coatings that have both anti-wear and anti-corrosion properties.

Nitrides, carbides, and oxides are promising materials for protective coatings against wear and corrosion. For instance, stainless steels coated with CrCN showed enhanced corrosion resistance and wear resistance [8]. TiN coatings greatly improved the wear resistance and corrosion resistance of stainless steel in simulated body fluid (SBF) solution [9,10]. Ti-O-N, SiO*_x_* and TiB*_x_*C*_y_*/a-C coatings are also candidates for potential applications in biomedical sectors due to their good corrosion resistance and wear resistance [11,12,13]. However, these single coatings suffered from severe delamination failure in corrosive media due to micro-crack propagation [13,14]. Recent results reported that coatings with duplex architecture could effectively prevent micro-crack propagation and such coatings showed better tribocorrosion performances compared to single component coatings [14,15,16,17], indicating a feasible strategy to design protective coatings for tribocorrosion applications.

Tribocorrosion is defined as degradation due to the combined effect of mechanical wear and electrochemical corrosion [18,19,20,21]. This synergistic damage is often more intense than wear and/or anodic dissolution alone [22]. Li et al. [23,24] reported that TiSiN and TiCN showed a higher wear rate in seawater than in air. Wang [14] reported that micro-cracks played an important role in determining the rate of delamination of a coating. However, the mechanism of damage due to the synergistic wear-corrosion effect is still not clearly known. Si element is an essential trace element for the normal growth and development of bone. Si-containing coatings show good prospects for application in biomedical implants (e.g., hip and knee). However, there are only few studies on the tribocorrosion failure mechanism of Si-containing coatings in a chloride ion-rich corrosive environment. Here a TiN/SiO*_x_* duplex coating was designed to enhance the wear resistance and corrosion resistance of stainless steel. The purpose of the TiN interlayer is to retard chloride ion corrosion and provide load support for the top layer of SiO*_x_*. The tribocorrosion failure behavior of the as-deposited coating was investigated in NaCl solution. The synergistic damage mechanism of wear-corrosion in the duplex coating is also discussed.

## 2. Materials and Methods

The TiN/SiO*_x_* duplex coating was synthesized using a multi-purpose plasma immersion ion implantation and deposition facility, where continuous plasma immersion ion implantation and deposition (PIIID) and radio frequency magnetron sputtering (RFMS) processes can be carried out without breaking vacuum [25,26,27]. The AISI304 stainless steel was used as the substrate. All the samples were mechanically polished using diamond paste (average particle size of 20 μm) followed by ultrasonic cleaning in pure ethanol. High purity Ti and Si were used as the targets. High purity Ar (99.999%), N_2_ (99.999%), and O_2_ (99.999%) were used to provide the working atmosphere. During the experiment, the vacuum chamber was evacuated to a base pressure of 0.005 Pa. Prior to the deposition of the coating, the polished samples were cleaned by Ar^+^ sputtering at a bias voltage of 6 kV for 30 min to remove residual pollution. The TiN layer was deposited in a N_2_ atmosphere. The experimental parameters were as follows: pressure 0.3 Pa, bias voltage 20 kV, frequency 75 Hz, bias voltage duration 60 µs, and deposition time 2 h. Subsequently, the SiO*_x_* layer was deposited in Ar and O_2_ atmosphere by RFMS with the following experimental parameters: pressure 2 Pa, bias voltage 6 kV, frequency 100 Hz, bias voltage duration 60 µs, RF power 400 W, and deposition time 2 h.

The crystalline structure of the coating was determined by X-ray diffraction (XRD, X’pert Powder, PANalytical, Almelo, The Netherlands) using a Cu Kα radiation source. The diffraction angle ranged from 20° to 90° and a scanning rate of 0.1°/s was used. The measurement was conducted with an incidence angle of 0.8°, a voltage of 40 kV, and a current of 40 mA. The scan step and integration time were 1400 and 0.5 s, respectively. The chemical bonding states of the component layers were detected by X-ray photoelectron spectroscopy (XPS, Thermo, K-Alpha, Waltham, MA, USA) using Al Kα radiation as the excitation source. Argon ion etching (accelerating voltage 1000 eV) was performed to remove contaminants. A field emission scanning electron microscopy (SEM, JSM-6701F, JEOL, Tokyo, Japan) was used to observe the cross-sectional morphology of the coating. The surface roughness of the coating was determined using an atomic force microscope (AFM, Dimension 3100, Veeco Instruments Inc., Plainview, NY, USA) in contact mode. The surface roughness (*Ra*) was obtained for a scanned area of 4 μm^2^. The mechanical properties of the component layers were evaluated by a nano-indentation system (Nano Indenter^®^ G200, Palo Alto, CA, USA). The hardness (*H*) and elastic modulus (*E*) were calculated using the Oliver-Pharr method [28]. The corrosion behavior of the coatings in 3.5 wt % NaCl solution was investigated electrochemically (PGSTAT302N, Herisau, Switzerland). High purity platinum was used as the counter electrode, the sample as the working electrode (1 cm^2^ exposed areas) and a saturated calomel electrode (SCE) as the reference electrode. The electrochemical tests were conducted in potentiostatic mode. The potential range and scanning rate were ±250 mV and 1 mV/s, respectively. Each test was repeated three times to ensure reproducibility.

**Figure 1:**
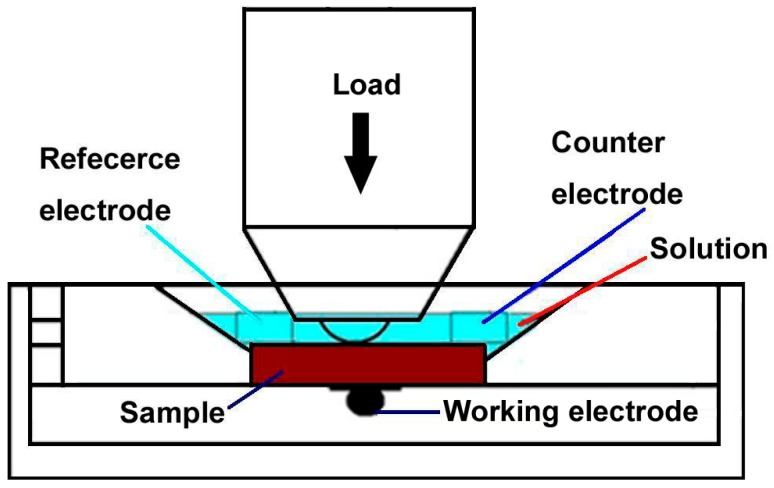
Scheme of the tribocorrosion apparatus.

A reciprocating sliding tribometer (MFT-R4000, Lanzhou Institute of Chemical Physics, Chinese Academy of Sciences, Lanzhou, China) was employed to evaluate the tribocorrosion failure behaviors of the coating in a 3.5 wt % NaCl solution. As shown in Figure 1, high purity graphite was used as the counter electrode, the sample as the working electrode and a saturated calomel electrode (SCE) as the reference electrode. During the tribocorrosion test, the open circuit potential (OCP) was recorded for 5 min prior to sliding after which the sliding test was continued for 15 min. A load of 5 N was applied on the sample using an Al_2_O_3_ ball (Φ 6 mm). The initial Hertzian contact pressure was about 0.987 GPa. The amplitude and frequency were 5 mm and 0.1 Hz, respectively. The OCP recording was continued for 5 min after the sliding had ended. The sliding test under atmosphere was also conducted under the same conditions for 15 min in order to investigate the wear-corrosion synergistic damage mechanisms. Each test was repeated three times under the same conditions in order to check the reproducibility. The “mean by cycle” values of the friction coefficients were used to evaluate the wear performance of the duplex coating. The wear track surface morphologies and the chemical composition of the coatings after the sliding tests were examined by SEM.

## 3. Results and Discussion

**Figure 2:**
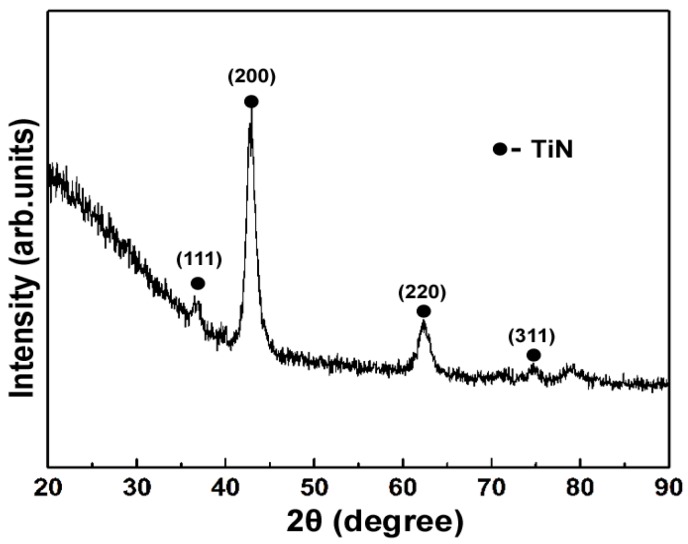
XRD pattern of the TiN/SiO*_x_* duplex coating.

Figure 2 shows the XRD pattern of the TiN/SiO*_x_* duplex coating. It can be seen that the Bragg refection peaks located at 36.71°, 42.63°, and 61.97° can be assigned to reflections from (111), (200) and (220) planes respectively, of face-centered cubic TiN [29]. No obvious diffraction peaks for silicon oxide phases were identified in this coating, indicating that the top SiO*_x_* layer is amorphous or poorly crystallized.

**Figure 3:**
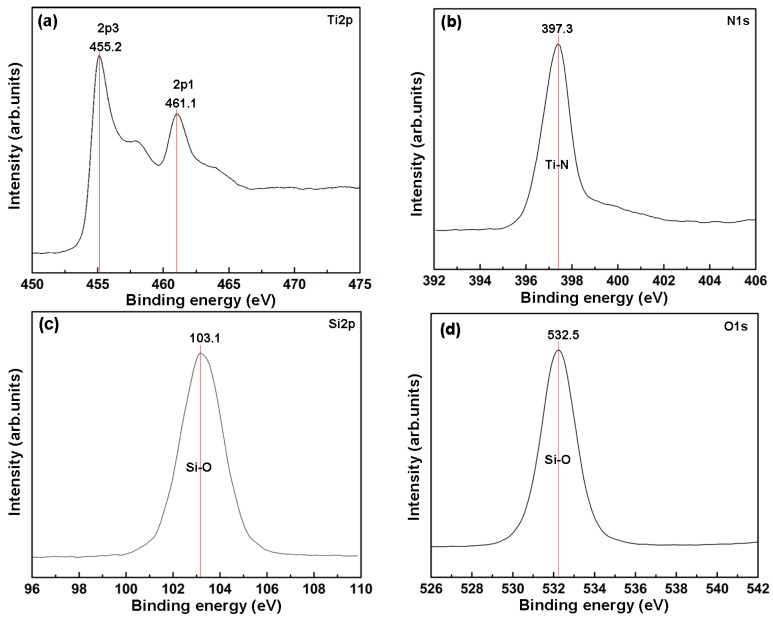
XPS spectrum of the TiN and SiO*_x_* component layers: (**a**) Ti2p; (**b**) N1s; (**c**) Si2p; (**d**) O1s.

Figure 3 presents the XPS spectrum of the TiN and SiO*_x_* component layers. As shown in Figure 3a,b, the binding energies of Ti2p3/2 and N1s peaks are at 455.2 eV and 397.3 eV, respectively, which are attributed to the presence of the TiN [30]. These results are in good agreement with the XRD characterization (see Figure 2). The binding energies of Si2p (Figure 3c) and O1s (Figure 3d) are centered at 103.1 eV and 532.5 eV, respectively, indicating that silicon in the coating is bound to oxygen [31].

**Figure 4:**
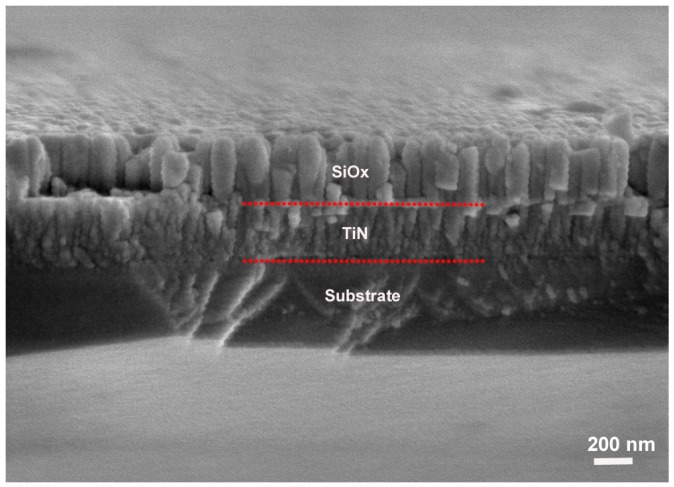
Cross-sectional SEM image of the TiN/SiO*_x_* duplex coating.

Figure 4 shows the cross-sectional SEM image of the TiN/SiO*_x_* duplex coating. A clear two-layered structure is observed and all the component layers exhibit typical columnar growth. However, the TiN interlayer consists of more densely packed and refined crystals compared to the SiO*_x_* layer. The thickness of the TiN and SiO*_x_* layers are 325 nm and 298 nm, respectively.

**Figure 5:**
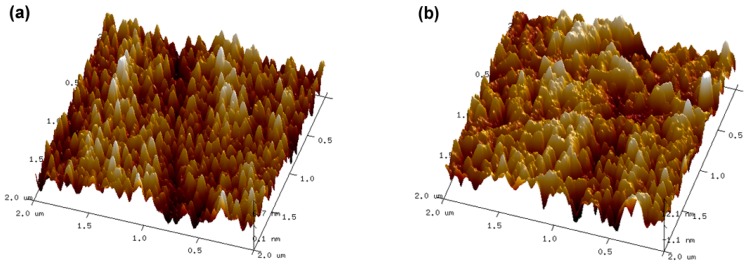
AFM surface morphologies of the component layers: (**a**) TiN interlayer; (**b**) SiO*_x_* layer.

Figure 5 shows the AFM surface morphologies of the component layers. As shown in Figure 5a, the surface roughness (*Ra*) of the TiN interlayer is 1.49 nm, indicating that this layer is smoother than that of the SiO*_x_* layer (Figure 5b, *Ra* = 2.77 nm).

**Figure 6:**
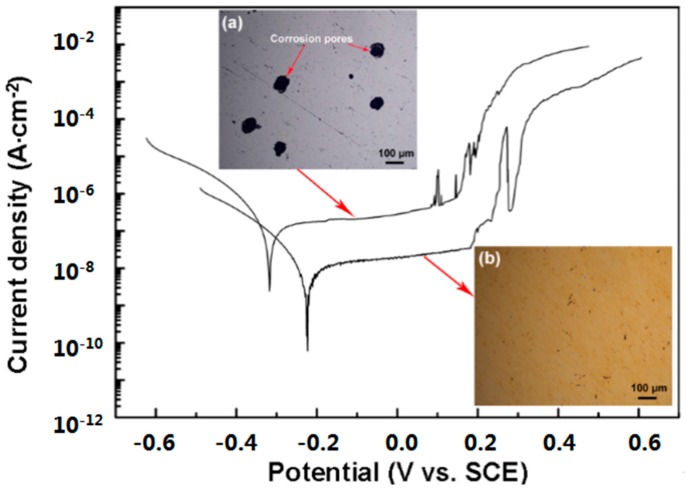
Anode polarization curves and corrosion morphologies of (**a**) substrate and (**b**) TiN/SiO*_x_* duplex coating.

Figure 6 shows the anode polarization curves and corrosion images of the substrate and TiN/SiO*_x_* duplex coating. As shown in Figure 6a, the corrosion potential and corrosion current density of the substrate are −317 ± 43 mV and 1.64 ± 0.31 × 10^−7^ A/cm^2^, respectively. The corresponding image reveals that a large number of corrosion pores are found distributed on the substrate surface. The TiN/SiO*_x_* duplex coating exhibits a better corrosion resistance with a higher corrosion potential of −272 ± 31 mV and a lower corrosion current density of 1.72 ± 0.49 × 10^−8^ A/cm^2^ (Figure 6b). This coating exhibits a smooth morphology and few corrosion defects are observed, indicating the excellent corrosion protection capability of the coating for the substrate.

**Figure 7:**
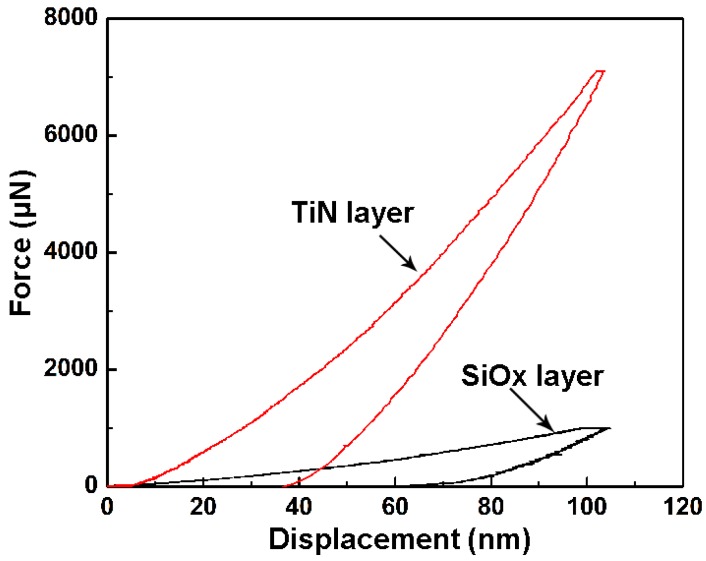
Nanoindentation curves of the component layers.

Figure 7 shows the nano-indentation curves of the component layers. The hardness, elastic modulus and *H*/*E* value of the TiN layer are 19.80 ± 0.35 GPa, 186.7 ± 9.54 GPa and 0.106, respectively. The SiO*_x_* layer shows a hardness of 5.2 ± 0.72 GPa, an elastic modulus of 110.9 ± 12.41 GPa and an *H*/*E* value of 0.047. Nano-indentation results confirm that the TiN interlayer exhibits better mechanical properties as compared to the SiO*_x_* layer.

**Figure 8:**
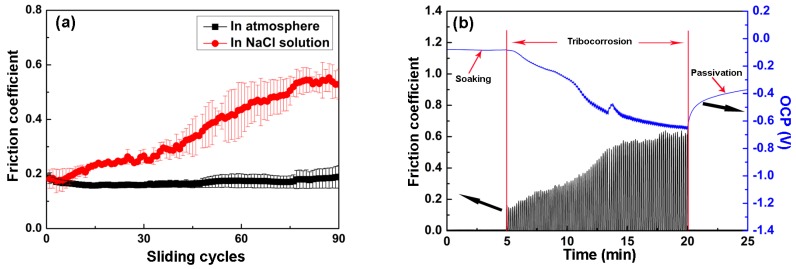
(**a**) Friction coefficient of the TiN/SiO*_x_* duplex coating; (**b**) a typical tribocorrosion curve with the OCP values of the TiN/SiO*_x_* duplex coating in NaCl solution.

Figure 8a presents the variation of the friction coefficient of the TiN/SiO*_x_* duplex coating during sliding tests. This coating exhibits a relatively stable wear performance in air with a constant friction coefficient of 0.18 during the whole sliding cycle. However, the coating shows an unstable wear performance in NaCl solution. The friction coefficient increases gradually and reaches the maximum value of 0.55 at the end of 80 sliding cycles. Figure 8b is a typical tribocorrosion curve showing the variation in OCP of the TiN/SiO*_x_* duplex coating in NaCl solution. It can be seen that this coating exhibits a relatively stable OCP value of −0.05 V during the soaking, but the OCP value decreases by 0.55 V during tribocorrosion and increases gradually to −0.38 V during passivation. Moreover, the decline in OCP value is proportional to the increase in frictional force, indicating the intense wear-corrosion synergistic effect.

**Figure 9:**
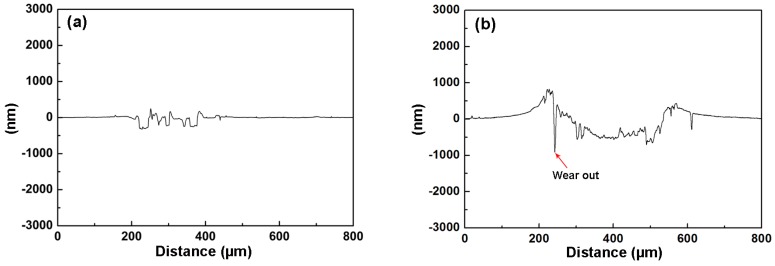
Cross-sectional profiles of the wear track after sliding test: (**a**) in air; (**b**) in NaCl solution.

Figure 9 shows the typical cross-sectional profiles of the wear track after the sliding test. When tested in air, the TiN/SiO*_x_* duplex coating exhibits a chunk peeling profile under atmosphere with a wear track width of 198 μm and a wear track depth of 294 nm (Figure 9a). The wear track profile shown in Figure 9b reveals that the TiN/SiO*_x_* duplex coating suffers from severe wear in NaCl solution and the wear track is wider (396 μm). The maximum wear track depth is up to 795 nm (marked by arrows). By referring to the corresponding component layer (see Figure 4), it can be inferred that the stable friction coefficient in air atmosphere involves only the SiO*_x_* layer. However, the unstable friction coefficient in NaCl solution indicates that both the substrate and the TiN interlayer are involved during tribocorrosion.

**Figure 10:**
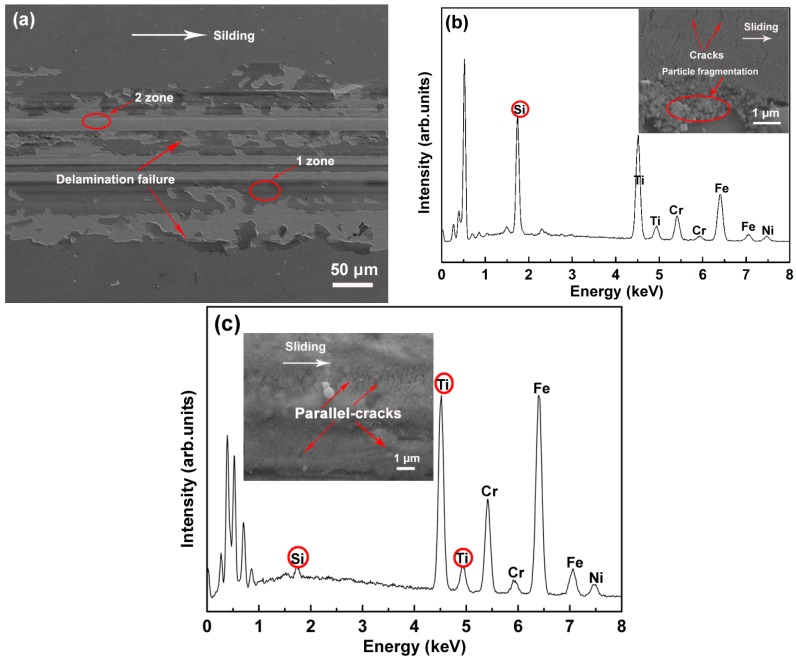
(**a**) SEM wear track image of the TiN/SiO*_x_* duplex coating in air; (**b**) EDS analysis of zone 1; (**c**) EDS analysis of zone 2.

Figure 10 shows the SEM image and energy dispersive spectroscopy (EDS) analysis of the wear track after sliding tests in air. The wear track shows a number of coating spallings and peelings (Figure 10a). The strong Si peak identified in zone 1 indicates that the peeling occurs in the SiO*_x_* layer (Figure 10b). The SEM image (insert in Figure 10b) reveals that the SiO*_x_* layer undergoes failure by particle fragmentation. EDS analysis of zone 2 reveals a strong Ti peak and a weak Si peak, confirming that the wear occurs in the TiN interlayer for this zone (Figure 10c). The high-resolution SEM image in Figure 10c shows that the wear track is characterized by a large number of parallel cracks.

**Figure 11:**
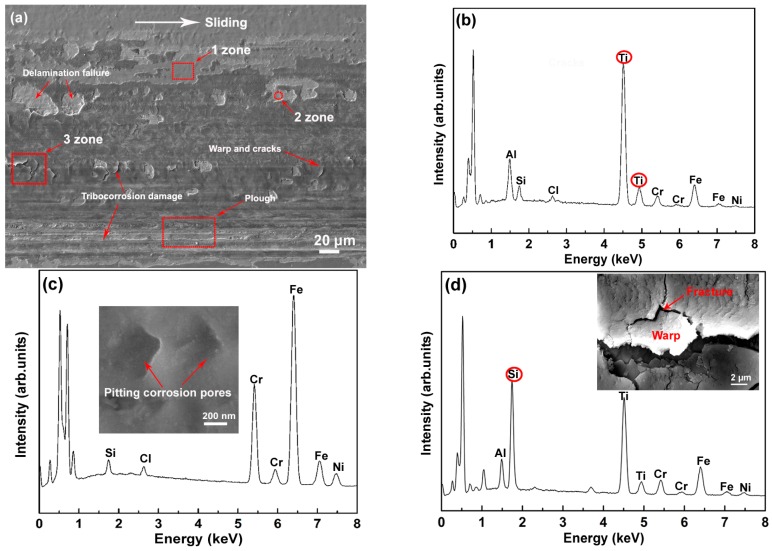
(**a**) SEM images of wear track in TiN/SiO*_x_* duplex coating in NaCl solution; (**b**) EDS analysis of zone 1; (**c**) EDS analysis of zone 2; (**d**) EDS analysis of zone 3.

Figure 11 shows the SEM image and EDS analysis of the wear track after the tribocorrosion test. It can be seen that severe coating delamination and tribocorrosion damages appear in the wear track (Figure 11a). The strong Ti peaks indentified in zone 1 confirm that the SiO*_x_* layer tends to delaminate from the TiN interlayer (Figure 11b). The EDS analysis of zone 2 reveals the presence of Fe, Cr, Ni, and Si, whereas Ti is absent in this zone (Figure 11c). These results imply that the delamination failure occurs at the interface of the TiN interlayer with the substrate. Micro-pores observed in the failure zone confirm the occurrence of severe pitting corrosion (SEM image inset in Figure 11c). The strong Si peak identified in zone 3 proves that the SiO*_x_* layer does not show wear failure, but undergoes intense tribocorrosion damage with severe warp and fracture (SEM image inset in Figure 11d). These failure characteristics are clearly different from those shown in Figure 10b.

**Figure 12:**
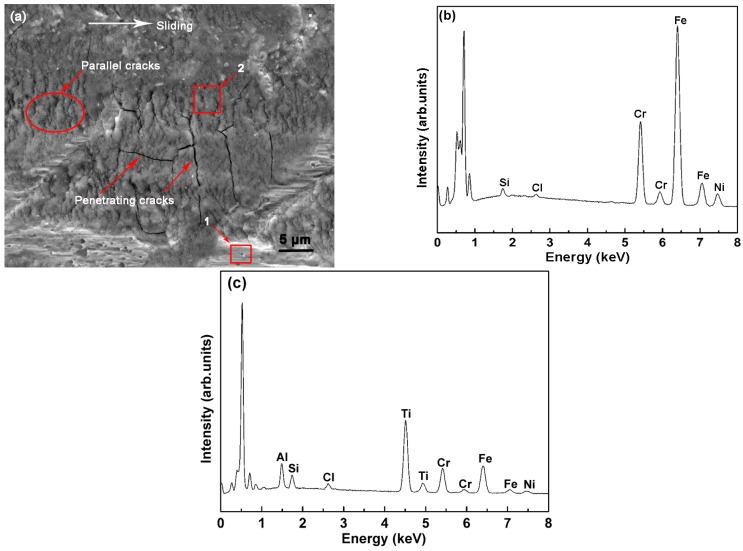
(**a**) High resolution SEM image of the tribocorrosion plough; (**b**) EDS analysis of zone 1; (**c**) EDS analysis of zone 2.

Figure 12 shows the high-resolution SEM image and EDS analysis of the plough after tribocorrosion test. A number of parallel and penetrating cracks are observed on the surface of the plough (Figure 12a). The presence of Fe, Cr, Ni, and Si in zone 1 indicates that the TiN/SiO*_x_* duplex coating has worn out (Figure 12b). The strong Ti peak identified in zone 2 conforms that the tribocorrosion occurs in the TiN interlayer (Figure 12c). The presence of penetrating cracks in this coating, in contrast to the wear track in air (Figure 10c), confirms that the TiN interlayer suffers more severe damage in NaCl solution.

The sliding test shows that the TiN interlayer exhibits excellent adhesion with the substrate, whereas the SiO*_x_* layer undergoes severe delamination failure. These failure characteristics can be attributed to the differences in the mechanical properties of the component layers. As shown in Figure 7, the *H*/*E* value of the TiN interlayer is 0.106, which is higher than that of the SiO*_x_* layer (0.047). It is generally believed that the ability of a coating to resist mechanical degradation and fracture is improved by high hardness, low elastic modulus and a high *H*/*E* ratio [32,33]. Therefore, the soft SiO*_x_* layer suffers from severe fracture failure due to its poor fracture resistance.

The tribocorrosion test confirms that the TiN/SiO*_x_* duplex coating shows a lower wear resistance in NaCl solution than in air. Plenty of penetrating cracks and layered delamination appear on the wear track (Figure 11). These multi-degradation failures mainly originate from force-corrosion synergy damage. As shown in Figure 4, this coating has a typical columnar growth structure, which benefits the initiation and propagation of micro-cracks along the columnar boundaries. Frictional force induces a large number of parallel cracks in the coating (see SEM images in Figure 10b,c). These micro-cracks provide channels and thereby accelerate the diffusion of NaCl solution. It has been reported that chloride ion plays a major role in inducing the pitting corrosion of materials [34,35]. The visible micro-pores and the presence of elemental Cl in the wear track confirm the simultaneous chloride ion corrosion during the tribocorrosion (see Figure 11c). This multi-scale chloride ion corrosion greatly degrades the bonding strength between the columnar crystals and the adjacent layers (e.g., substrate/TiN and TiN/SiO*_x_*). Consequently, the intense force-corrosion synergy interaction leads to a severe multi-degradation failure of the TiN/SiO*_x_* duplex coating. 

## 4. Conclusions

This study investigated the tribocorrosion failure behavior of TiN/SiO*_x_* duplex coatings in NaCl solution; the main conclusions are as follows:

(1). The TiN/SiO*_x_* duplex coating has a two-layered columnar growth structure which is made up of a face-centered cubic TiN interlayer and a layer of amorphous SiO*_x_*, and provides excellent corrosion protection to the stainless steel substrate.
(2). The TiN interlayer exhibits good adhesion with the substrate, but the SiO*_x_* layer suffers from severe delamination failure during the sliding test in air.
(3). Frictional force induces a large number of micro-cracks in the TiN/SiO*_x_* duplex coating. These micro-cracks provide diffusion channels for the NaCl solution and eventually cause simultaneous chloride ion corrosion during the tribocorrosion.
(4). The multi-scale Cl ion corrosion greatly weakens the bonding strength of the columnar crystals or the adjacent layers. The force-corrosion synergy interaction damage induces multi-degradation failures in the TiN/SiO*_x_* duplex coating.

Our results confirm that synergistic wear-corrosion damage greatly accelerates the failure of the duplex coating. It is therefore important to choose coating materials that have a high *H*/*E* ratio as well as a dense structure to design future biomedical duplex coatings.
